# Supplementary material for: SEPepQuant enhances the detection of possible isoform regulations in shotgun proteomics
Source: Nat Commun. 2023 Sep 19;14:5809. doi: 10.1038/s41467-023-41558-2 (PMC10509223; doi:10.1038/s41467-023-41558-2)
Supplement: Supplementary file 1 — Supplementary Information [file 41467_2023_41558_MOESM1_ESM.pdf]

SEPepQuant enhances the detection of possible isoform regulations in shotgun proteomics

Yongchao Dou[1,2], Yuejia Liu[3], Xinpei Yi[1,2], Lindsey K. Olsen[1,2], Hongwen Zhu[4], Qiang Gao[5], Hu Zhou[3,4], Bing Zhang[1,2#]

[1]Lester and Sue Smith Breast Center, Baylor College of Medicine, Houston, TX 77030, USA

[2]Department of Molecular and Human Genetics, Baylor College of Medicine, Houston, TX 77030, USA

[3]School of Chinese Materia Medica, Nanjing University of Chinese Medicine, 138 Xianlin Avenue, Nanjing, Jiangsu 210023, China

[4]Department of Analytical Chemistry, State Key Laboratory of Drug Research and CAS Key Laboratory of Receptor Research, Shanghai Institute of Materia Medica, Chinese Academy of Sciences, 555 Zuchongzhi Road, Shanghai 201203, China

[5]Department of Liver Surgery and Transplantation, Liver Cancer Institute, Zhongshan Hospital, Fudan University, and Key Laboratory of Carcinogenesis and Cancer Invasion of Ministry of Education, 180 Fenglin Road, Shanghai 200032, China

# Correspondence should be addressed to B.Z. (bing.zhang@bcm.edu)

Supplementary Information

Supplemental figure 1: Summary of existing methods and classification of the *in silico* digested peptides with 1 or 2 miscleavages or semi-tryptic peptides.

Supplemental figure 2: SEPEP level quality control

Supplemental figure 3: Evaluation of SEPepQuant on an iPSC data set

Supplemental figure 4: Evaluation of SEPepQuant on two liver cancer data sets

Supplementary Dataset 1: Percentage of peptides with missed cleavage site(s)

Supplementary Dataset 2: iPSC-TMT SEPEP quantification, median centered

Supplementary Dataset 3: iPSC-TMT SEPEP mapping table

Supplementary Dataset 4: iPSC-FragPipe quantification

Supplementary Dataset 5: HCC-TMT SEPEP quantification, median centered

Supplementary Dataset 6: HCC-TMT SEPEP mapping table

Supplementary Dataset 7: HCC-TMT FragPipe quantification

Supplementary Dataset 8: HCC-label free SEPEP PSM count

Supplementary Dataset 9: HCC-label free SEPEP mapping table

Supplementary Dataset 10: HCC-label free FragPipe quantification

Supplementary Dataset 11: iPSC-TMT gene and SEPEP correlation with culture time

Supplementary Dataset 12: HCC-TMT gene and SEPEP tumor versus normal comparison

Supplementary Dataset 13: HCC-TMT gene and SEPEP survival analysis

Supplementary Dataset 14: Overlapping significant genes from the HCC-TMT tumor versus normal and survival analyses, as well as information and results from the PRM analysis.

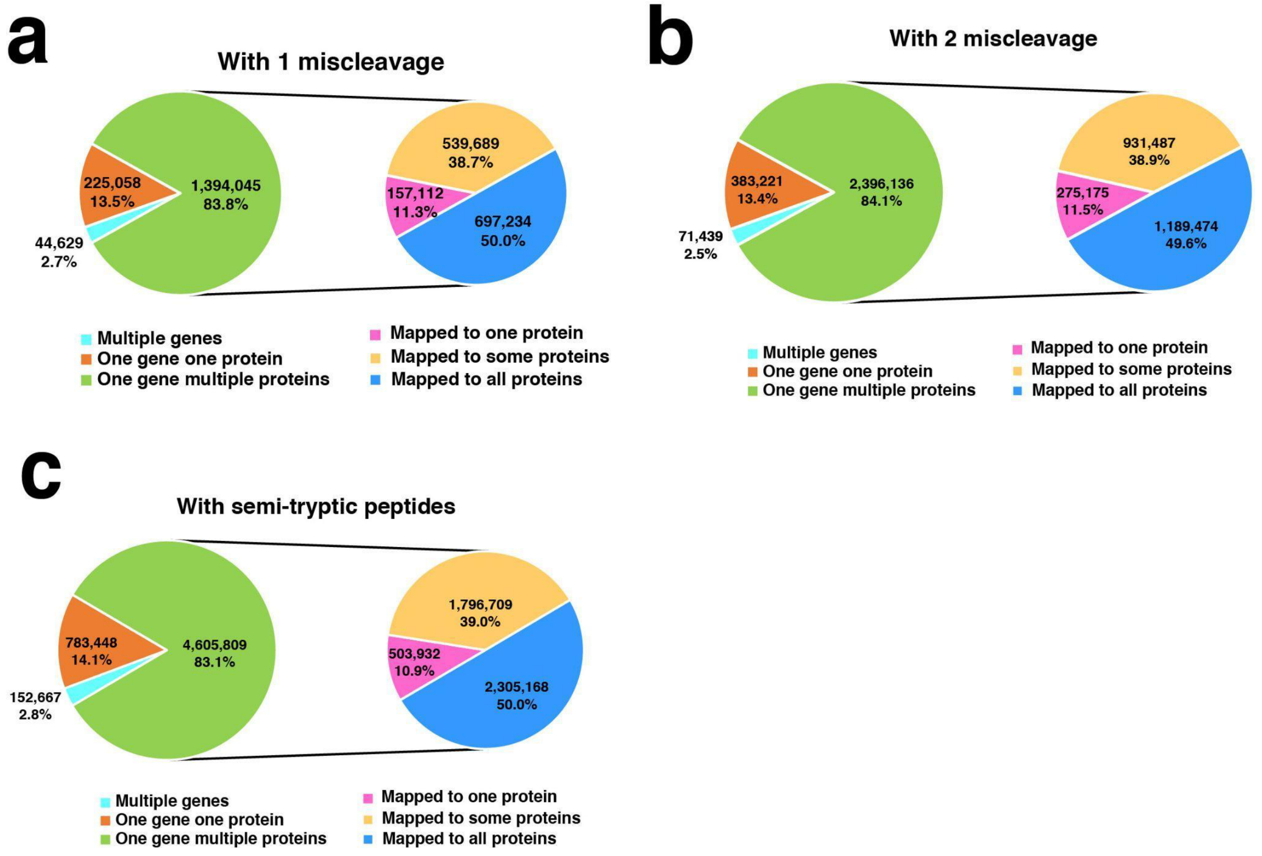

Supplemental Figure 1. Classification of the *in silico* digested peptides with 1 (a) and 2 (b) miscleavages and semi-tryptic peptides (c) based on their mapping to genes and protein isoforms.

a
b

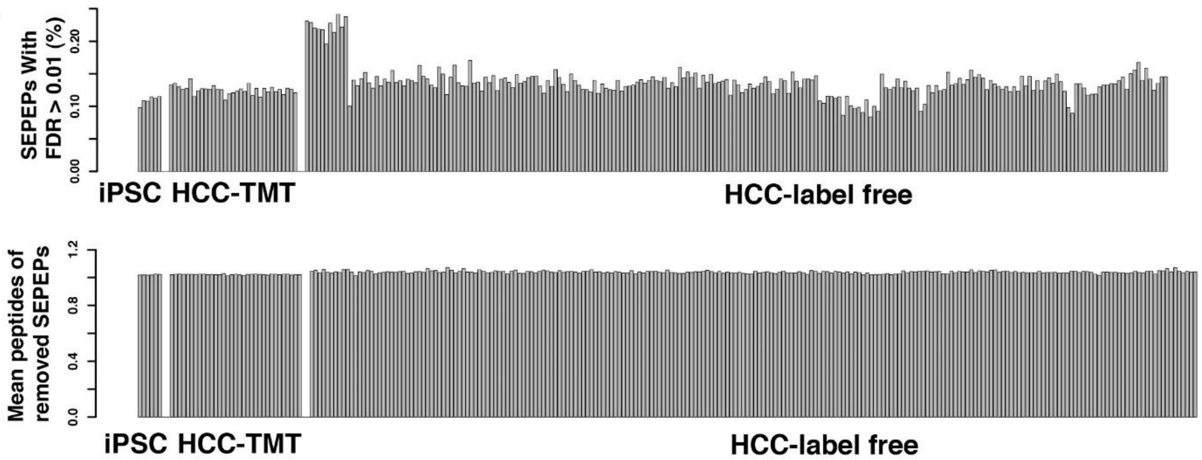

c

		iPSC-MaxQuant	
		SEPEP-based	Protein group
	Gene with multiple features	861	20
Feature distribution	2	638	18
	3	170	1
	4	33	0
	5	13	0
	>=6	20	1

d

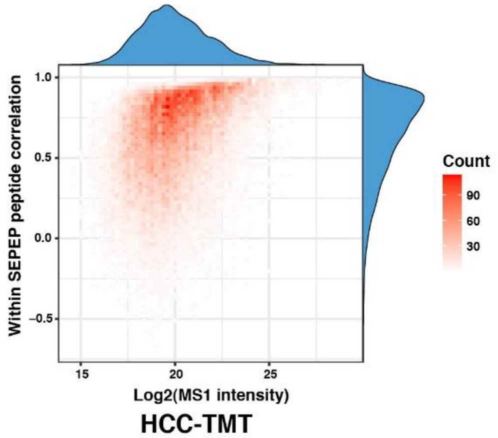

Supplemental figure 2: SEPEP level quality control. (a) Percentages of SEPEPs with FDR >0.01. (b) Mean peptide numbers of SEPEPs with FDR >0.01. (c) Comparison of numbers of genes with multiple SEPEPs in SEPepQuant analysis and those with multiple protein groups by MaxQuant. (d) A density plot showing the distribution of within-SEPEP peptide correlations and their relationship with average MS1 peptide intensities. Source data of s2a, s2b, and s2d are provided as a Source Data file.

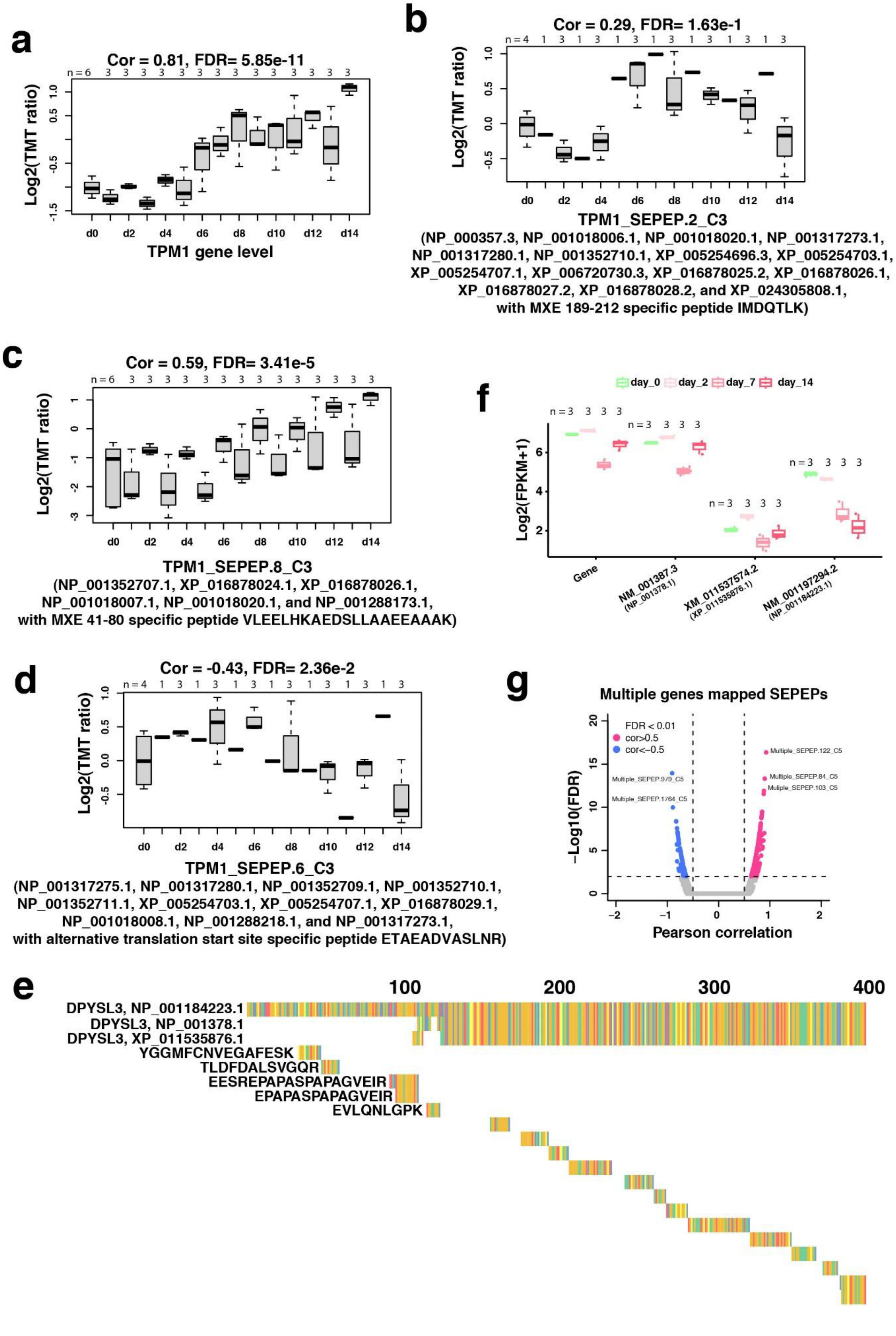
a
Cor = 0.81, FDR= 5.85e-11
Log2(TMT ratio)
d0 d2 d4 d6 d8 d10 d12 d14
TPM1 gene level
b
Cor = 0.29, FDR= 1.63e-1
Log2(TMT ratio)
d0 d2 d4 d6 d8 d10 d12 d14
TPM1_SEPEP.2_C3
(NP_000357.3, NP_001018006.1, NP_001018020.1, NP_001317273.1,
NP_001317280.1, NP_001352710.1, XP_005254696.3, XP_005254703.1,
XP_005254707.1, XP_006720730.3, XP_016878025.2, XP_016878026.1,
XP_016878027.2, XP_016878028.2, and XP_024305808.1,
with MXE 189-212 specific peptide IMDQTLK)
c
Cor = 0.59, FDR= 3.41e-5
Log2(TMT ratio)
d0 d2 d4 d6 d8 d10 d12 d14
TPM1_SEPEP.8_C3
(NP_001352707.1, XP_016878024.1, XP_016878026.1,
NP_001018007.1, NP_001018020.1, and NP_001288173.1,
with MXE 41-80 specific peptide VLEELHKAEDSLLAAEEAAAK)
f
day_0 day_2 day_7 day_14
Log2(FPKM+1)
Gene
NM_001387.3 (NP_001378.1)
XM_011537574.2 (XP_011535876.1)
NM_001197294.2 (NP_001184223.1)
d
Cor = -0.43, FDR= 2.36e-2
Log2(TMT ratio)
d0 d2 d4 d6 d8 d10 d12 d14
TPM1_SEPEP.6_C3
(NP_001317275.1, NP_001317280.1, NP_001352709.1, NP_001352710.1,
NP_001352711.1, XP_005254703.1, XP_005254707.1, XP_016878029.1,
NP_001018008.1, NP_001288218.1, and NP_001317273.1,
with alternative translation start site specific peptide ETAEADVASLNR)
g
Multiple genes mapped SEPEPs
FDR < 0.01
cor>0.5
cor<-0.5
Multiple_SEPEP.122_C5
Multiple_SEPEP.84_C5
Mulitple_SEPEP.103_C5
Multiple_SEPEP.979_C5
Multiple_SEPEP.1764_C5
−Log10(FDR)
Pearson correlation
e
100 200 300 400
DPYSL3, NP_001184223.1
DPYSL3, NP_001378.1
DPYSL3, XP_011535876.1
YGGMFCNVEGAFESK
TLDFDALSVGQR
EESREPAPASPAPAGVEIR
EPAPASPAPAGVEIR
EVLQNLGPK

Supplemental figure 3: Evaluation of SEPepQuant on an iPSC data set. (a-d) Protein abundance and culture time correlations of TPM1 gene and selected SEPEPs. (e) Identified peptides of DPYSL3 on iPSC data set from 1- 400bp. (f) Gene and transcript isoform expression of DPYSL3 in RNASeq data. (g) Correlations between cell culture time and multi-genes SEPEPs. The p-values were calculated using Pearson's Correlation and the Benjamini and Hochberg method was used to adjust p-values for multiple comparisons. For boxplots, centerline indicates the median, box limits indicate upper and lower quartiles, whiskers indicate the 1.5 interquartile range. Source data of s3a, s3b, s3c, s3d, s3f, and s3g are provided as a Source Data file.

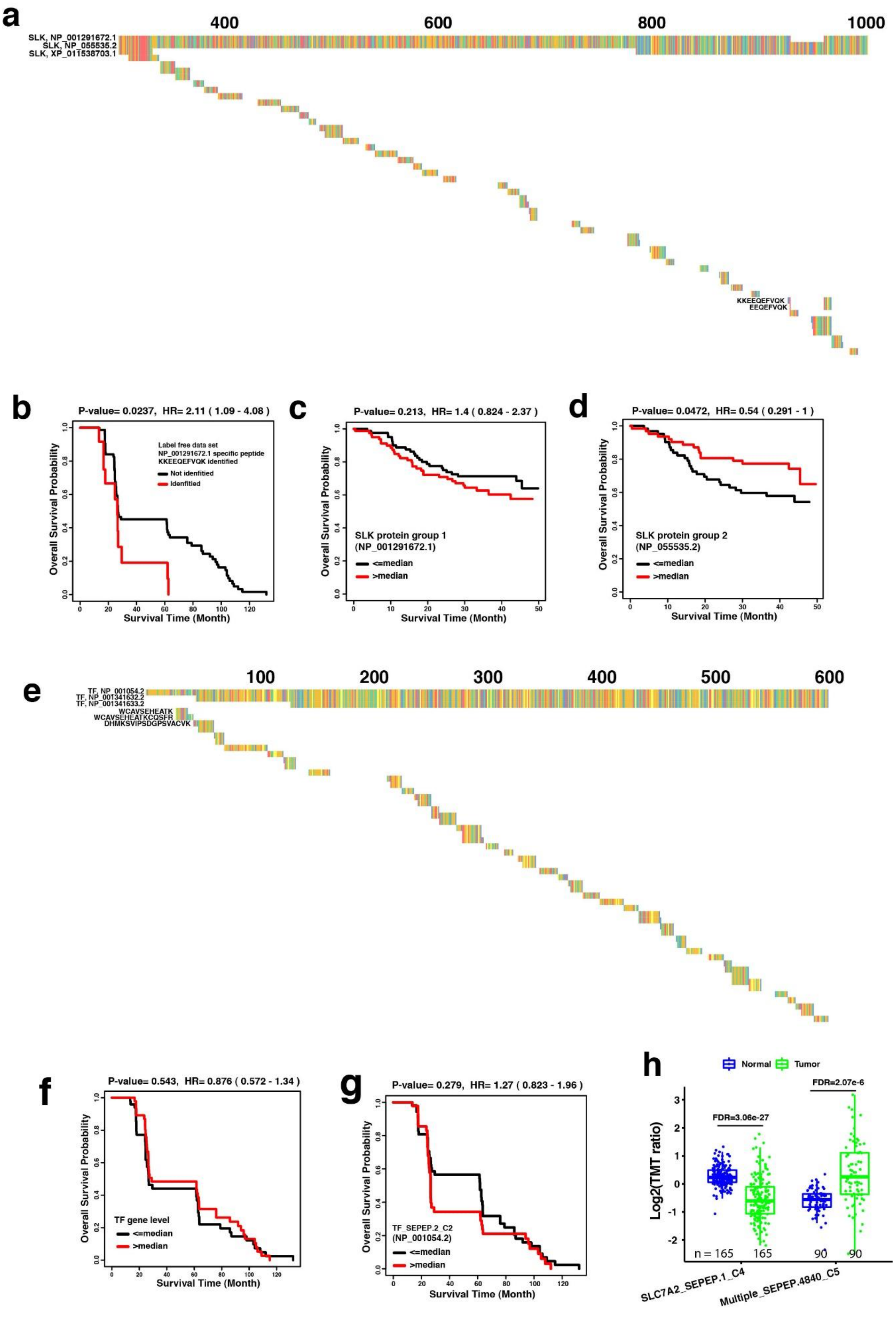

a
400
600
800
1000
SLK, NP_001291672.1
SLK, NP_055535.2
SLK, XP_011538703.1
KKEEQEFVQK
EEQEFVQK
b
P-value= 0.0237, HR= 2.11 (1.09 - 4.08)
Label free data set
NP_001291672.1 specific peptide
KKEEQEFVQK identified
Not idenfitied
Idenfitied
Overall Survival Probability
Survival Time (Month)
c
P-value= 0.213, HR= 1.4 (0.824 - 2.37)
SLK protein group 1
(NP_001291672.1)
<=median
>median
d
P-value= 0.0472, HR= 0.54 (0.291 - 1)
SLK protein group 2
(NP_055535.2)
<=median
>median
e
100
200
300
400
500
600
TF, NP_001054.2
TF, NP_001341632.2
TF, NP_001341633.2
WCAVSEHEATK
WCAVSEHEATKCQSFR
DHMKSVIPSDGPSVACVK
f
P-value= 0.543, HR= 0.876 (0.572 - 1.34)
TF gene level
<=median
>median
g
P-value= 0.279, HR= 1.27 (0.823 - 1.96)
TF_SEPEP.2_C2
(NP_001054.2)
<=median
>median
h
Normal
Tumor
FDR=3.06e-27
FDR=2.07e-6
Log2(TMT ratio)
n = 165
165
90
90
SLC7A2_SEPEP.1_C4
Multiple_SEPEP.4840_C5

Supplemental figure 4: Evaluation of SEPepQuant on two liver cancer data sets. (a) Identified peptides of SLK on HCC-TMT data set. (b) Survival comparison between samples with and without NP_001291672.1 specific peptide KKEEQEFVQK on HCC-label free data set. (c) Correlation of NP_001291672.1 abundance by FragPipe and survival on HCC-TMT data set. (d) Correlation of NP_055535.2 abundance by FragPipe and survival on HCC-TMT data set. (e) Identified peptides of TF on HCC-TMT data set. (f-g) TF gene level and TF_SEPEP.2_C2 abundance and survival correlations on HCC-label free data set. (h) Tumor versus normal comparisons based on Multiple_SEPEP.4840_C5 and SLC7A2_SEPEP.1_C4 abundance, respectively. For boxplots, p-values were calculated using two-sided Student's t-test, the Benjamini and Hochberg method was used to adjust p-values for multiple comparisons, centerline indicates the median, box limits indicate upper and lower quartiles, whiskers indicate the 1.5 interquartile range. For survival analysis, p-values were calculated using Kaplan-Meier test. Source data of s4b, s4c, s4d, s4f, s4g, and s4h are provided as a Source Data file.
