# Supplementary material for: SEPepQuant enhances the detection of possible isoform regulations in shotgun proteomics
Source: Nat Commun. 2023 Sep 19;14:5809. doi: 10.1038/s41467-023-41558-2 (PMC10509223; doi:10.1038/s41467-023-41558-2)
Supplement: Supplementary file 3 — Description of Additional Supplementary Files [file 41467_2023_41558_MOESM3_ESM.pdf]

Description of Additional Supplementary Files:

Supplementary Dataset 1: Percentage of peptides with missed cleavage site(s)

Supplementary Dataset 2: iPSC-TMT SEPEP quantification, median centered

Supplementary Dataset 3: iPSC-TMT SEPEP mapping table

Supplementary Dataset 4: iPSC-FragPipe quantification

Supplementary Dataset 5: HCC-TMT SEPEP quantification, median centered

Supplementary Dataset 6: HCC-TMT SEPEP mapping table

Supplementary Dataset 7: HCC-TMT FragPipe quantification

Supplementary Dataset 8: HCC-label free SEPEP PSM count

Supplementary Dataset 9: HCC-label free SEPEP mapping table

Supplementary Dataset 10: HCC-label free FragPipe quantification

Supplementary Dataset 11: iPSC-TMT gene and SEPEP correlation with culture time

Supplementary Dataset 12: HCC-TMT gene and SEPEP tumor versus normal comparison

Supplementary Dataset 13: HCC-TMT gene and SEPEP survival analysis

Supplementary Dataset 14: Overlapping significant genes from the HCC-TMT tumor versus normal and survival analyses, as well as information and results from the PRM analysis.
