# Supplementary material for: SEPepQuant enhances the detection of possible isoform regulations in shotgun proteomics
Source: Nat Commun. 2023 Sep 19;14:5809. doi: 10.1038/s41467-023-41558-2 (PMC10509223; doi:10.1038/s41467-023-41558-2)
Supplement: Supplementary file 18 — Reporting Summary [file 41467_2023_41558_MOESM18_ESM.pdf]

nature portfolio

Corresponding author(s): Bing Zhang

Last updated by author(s): Aug 10, 2023

Reporting Summary

Nature Portfolio wishes to improve the reproducibility of the work that we publish. This form provides structure for consistency and transparency in reporting. For further information on Nature Portfolio policies, see our Editorial Policies and the Editorial Policy Checklist.

Statistics

For all statistical analyses, confirm that the following items are present in the figure legend, table legend, main text, or Methods section.

n/a	Confirmed	
☐	☒	The exact sample size (n) for each experimental group/condition, given as a discrete number and unit of measurement
☐	☒	A statement on whether measurements were taken from distinct samples or whether the same sample was measured repeatedly
☐	☒	The statistical test(s) used AND whether they are one- or two-sided<br>*Only common tests should be described solely by name; describe more complex techniques in the Methods section.*
☒	☐	A description of all covariates tested
☒	☐	A description of any assumptions or corrections, such as tests of normality and adjustment for multiple comparisons
☐	☒	A full description of the statistical parameters including central tendency (e.g. means) or other basic estimates (e.g. regression coefficient) AND variation (e.g. standard deviation) or associated estimates of uncertainty (e.g. confidence intervals)
☐	☒	For null hypothesis testing, the test statistic (e.g. F, t, r) with confidence intervals, effect sizes, degrees of freedom and P value noted<br>*Give P values as exact values whenever suitable.*
☒	☐	For Bayesian analysis, information on the choice of priors and Markov chain Monte Carlo settings
☒	☐	For hierarchical and complex designs, identification of the appropriate level for tests and full reporting of outcomes
☐	☒	Estimates of effect sizes (e.g. Cohen's d, Pearson's r), indicating how they were calculated

Our web collection on statistics for biologists contains articles on many of the points above.

Software and code

Policy information about availability of computer code

Data collection	ProteoWizard pwiz-bin-windows-x86_64-vc143 was used to transfer raw files to mzML files.
Data analysis	The comprehensive shotgun proteomics analysis pipeline FragPipe (https://fragpipe.nesvilab.org/; V17.0) with MSFragger (v3.4) and philosopher (v4.2.1) was used for database searching and protein group level and gene level quantification for all three data sets. MaxQuant (v2.3.1.0) was only applied to the iPSC data set. SEPepQuant (v1.0.0-alpha) was used to do SEPEP level quantification which is available at https://github.com/bzhanglab/SEPepQuant. Protein sequences for genes with multiple protein isoforms were aligned using clustalw2 (v2.1) with default parameters. Then, clustalo (v1.2.4) was applied to above multiple sequence alignments to calculate sequence similarity with percent identities as sequence distance.

For manuscripts utilizing custom algorithms or software that are central to the research but not yet described in published literature, software must be made available to editors and reviewers. We strongly encourage code deposition in a community repository (e.g. GitHub). See the Nature Portfolio guidelines for submitting code & software for further information.

Data

Policy information about availability of data

All manuscripts must include a data availability statement. This statement should provide the following information, where applicable:

- Accession codes, unique identifiers, or web links for publicly available datasets
- A description of any restrictions on data availability
- For clinical datasets or third party data, please ensure that the statement adheres to our policy

The human iPSC and HCC-label free data sets were downloaded from PRIDE (www.ebi.ac.uk/pride) with accession numbers PXD013426 and PXD006512. The HCC-TMT proteome data was downloaded from NODE (https://www.biosino.org/node) by accession number OEP000321. Processed PRM data are available in Table S5. Other processed quantification data and downstream analysis results were included in Supplementary Tables 1-14. RefSeq gene annotation and matched protein database with all NP, XP, and YP protein sequences were downloaded on 06/28/2020.

Research involving human participants, their data, or biological material

Policy information about studies with human participants or human data. See also policy information about sex, gender (identity/presentation), and sexual orientation and race, ethnicity and racism.

Reporting on sex and gender	N/A
Reporting on race, ethnicity, or other socially relevant groupings	N/A
Population characteristics	N/A
Recruitment	N/A
Ethics oversight	N/A

Note that full information on the approval of the study protocol must also be provided in the manuscript.

Field-specific reporting

Please select the one below that is the best fit for your research. If you are not sure, read the appropriate sections before making your selection.

☒ Life sciences ☐ Behavioural & social sciences ☐ Ecological, evolutionary & environmental sciences

For a reference copy of the document with all sections, see nature.com/documents/nr-reporting-summary-flat.pdf

Life sciences study design

All studies must disclose on these points even when the disclosure is negative.

Sample size	Three published data sets were selected to cover different sample types and proteomics platforms. The iPSC data set is a human cell line data set generated on a 10-plex TMT platform. In this study, the human iPSC cells were cultured over 14 days and harvested daily for proteomics analyzing. The HCC TMT data set includes 165 tumor samples from hepatocellular carcinoma patients and matched adjacent normal tissues, and the data were generated on a 11-plex TMT plagrom. The HCC label-free data set includes 111 tumor samples from hepatocellular carcinoma patients and matched adjacent normal tissues, and the data were generated on a label-free platform.
Data exclusions	None
Replication	Twenty samples from the original liver cancer TMT study, 10 with good prognosis and 10 with bad prognosis, were analyzed by PRM to confirm our findings by TMT data. Five peptides were analyzed by PRM and their abundance can be found in Table S5.
Randomization	We did not need to use randomization in this study as this is a protein abundance quantification study and we used all of collected data.
Blinding	Blinding is not relevant to our study because it is not a testing of specific treatment conditions.

Reporting for specific materials, systems and methods

We require information from authors about some types of materials, experimental systems and methods used in many studies. Here, indicate whether each material, system or method listed is relevant to your study. If you are not sure if a list item applies to your research, read the appropriate section before selecting a response.

Materials & experimental systems

n/a	Involved in the study
☒	☐ Antibodies
☒	☐ Eukaryotic cell lines
☒	☐ Palaeontology and archaeology
☒	☐ Animals and other organisms
☒	☐ Clinical data
☒	☐ Dual use research of concern
☒	☐ Plants

Methods

n/a	Involved in the study
☒	☐ ChIP-seq
☒	☐ Flow cytometry
☒	☐ MRI-based neuroimaging
